# Study on compaction characteristics and mechanical model of dry crushing filling material under lateral confinement condition

**DOI:** 10.1038/s41598-024-65543-x

**Published:** 2024-06-28

**Authors:** Youlin Xu, Shaokang Wu, Zhisong Chen, Xukun Wu, Jitao Zhang, Bing Xiao

**Affiliations:** 1 School of Mining Engineering, Guizhou Institute of Technology, Guizhou, 550003 Guiyang China; 2 School of Energy and Mining Engineering, China University of Mining and Technology (Beijing), Beijing, 100083 China; 3 School of Mining, Guizhou University, Guizhou, 550025 Guiyang China; 4 School of Mining Engineering, Anhui University of Science and Technology, Huainan, 232001 Anhui China; 5 Guizhou Qianchenglijin Technology Co., Ltd, Guizhou, 550081 Guiyang China; 6 School of Resource & Environment and Safety Engineering, Hunan University of Science and Technology, Xiangtan, 411201 China

**Keywords:** Goaf, Dry filling material, Compaction characteristics, Load-bearing performance, Talbot coefficient, Acoustic emission, Natural hazards, Diseases, Risk factors, Energy science and technology, Engineering

## Abstract

The compaction characteristics and bearing capacity of dry filling materials in goaf have a significant influence on stope control and surface stability. Through acoustic emission monitoring and mechanical model analysis, a series of confined compression tests were conducted on crushed waste with varying particle sizes and Talbot coefficients. The deformation, fragmentation, and acoustic emission characteristics under corresponding working conditions were determined. The results indicate that the stress–strain curves of crushed stone with different particle sizes and Talbot coefficients exhibit similar nonlinear behavior during confined compression. However, the strain response varies with changing stress levels. By analyzing the slope change rate of the stress–strain curve, the lateral uniaxial compression process of waste rock can be divided into three deformation stages: rapid compression, stable crushing, and slow compaction. The compressive deformation characteristics of gravel differ based on particle size and Talbot coefficient. Specimens with a higher Talbot coefficient demonstrate stronger compressive resistance and weaker deformation resistance during initial compaction loading. Notably, the internal pressure structure strength is influenced by factors such as maximum particle size D, grading coefficient n, and particle size distribution continuity, rather than solely by the proportion of large particles. The evolution of acoustic emission signals and energy-time curve during waste rock confined axial compression synchronizes with the compaction process. Overall, compaction plays a critical role in maintaining the stability of goaf in dry crushed waste filling.

## Introduction

To facilitate the rapid growth of the industry, our country has been increasing its demand for mineral resources, resulting in a continuous escalation of domestic mineral resource extraction^1^. As mining activity intensifies, coal resources are increasingly being extracted from deeper areas^2^. Resulting in frequent geological disasters such as severe surface subsidence and landslides in mining areas. Consequently, addressing gob treatment has become an urgent challenge^3^. Backfill mining is a critical technique that entails filling solid waste materials into goafs to maintain stope and surface stability. While using cement or other cementing agents as filling materials can be costly, dry filling presents advantages such as lower costs and higher filling efficiency. As a result, dry filling has become a key area of focus in current research on backfill mining^4,5^. Therefore, studying the compaction deformation characteristics of dry crushed filling materials and the bearing properties after goaf filling holds significant importance^6^.

Currently, substantial advancements have been made in monitoring the factors that influence the compaction characteristics of bulk filling materials, as well as observing both macro and micro changes after compaction. Yang Ke et al.^7^ conducted compression tests on crushed gangue material with varying particle sizes, axial stresses, and loading rates to explore the bearing characteristics and rupture evolution law of gangue aggregate. Zhang Junwen et al.^8^ utilized self-developed large particle size grading and trapezoidal grading to conduct normal distribution loading tests, establishing the relationship between axial deformation of broken rock, residual crushing expansion coefficient, porosity, and axial load—a significant contribution to studying the bearing deformation characteristics of large-sized broken rock. Le et al^9^ designed lateral confined concentrated compression tests for various dry filling materials, comparing and analyzing numerical simulation results with test results to reveal the effects of particle size, gob height, and burial depth on surface subsidence. Pang et al.^10^ designed continuous and intermittent classification of two different structures, examined the particle size distribution of block coal gangue, determined the interaction between compaction and re-crushing, and established the compact-re-crushing model of crushed gangue. Yu Qunding et al.^11^ based on the particle stacking theory, investigated compaction and strength characteristics under seven different rock-sand ratios (G/S), concluding that optimal parameters such as dry density, shear strength, modulus of resilience, damping ratio, and cumulative plastic deformation were achieved when G/S = 1.6–1.8. Zhang Zongtang et al.^12^ determined the optimal grading interval of coal gangue subgrade packing through large-scale vibration compaction tests and large-scale triaxial tests. Liu et al.^13^ found a positive correlation between the crushing rate of gravel and the intermediate principal stress ratio through true triaxial compression tests. Wang Qiong et al.^14^ conducted trapezoid-shaped graded loading compression tests on gravel rock samples, obtaining the bearing deformation characteristics of crushed rock on the gravel side under the condition of cutting top pressure relief and self-forming lane. Mao et al.^15^ proposed a method that used acoustic emission to monitor the crushing process of single particles. According to the activity and frequency characteristics of acoustic emission signals, it was concluded that the gravel would have high-frequency AE aggregation before fracture failure. Lastly, Michlmayr et al.^16^, to study how microstructure reflects the influence of macroscopic stress–strain behavior, monitored the shear process of granular materials through acoustic emission and derived relevant laws.

In the studies mentioned above, the focus on the compaction characteristics and load-bearing performance of dry filling materials primarily revolves around examining the impact of parameters such as different particle sizes, loading rates, stress levels, and grading coefficients (n) on compaction characteristics. However, there has been limited exploration of the introduction of an acoustic emission monitoring system to analyze the compaction characteristics of crushed gangue under varying particle sizes and Talbot coefficients. Research on AE characteristics has mainly focused on brittle engineering materials like rock, concrete, granular soil, and granular materials, with little investigation into the AE characteristics of gob filled with crushed gangue, leading to an unclear understanding of the evolution law of internal microstructure and load-bearing characteristics of crushed gangue after filling^17,18^. This paper delves into the compression and deformation characteristics of gob broken rock through lateral confined compression-acoustic emission monitoring tests under different particle sizes and Talbot coefficients. Leveraging the acoustic emission monitoring system, the study investigates the evolution law of slip and rupture in the compaction process of gob broken rock, aiming to unveil the compressive deformation mechanism of gob broken gangue at the meso-texture level. Lastly, based on the experimental research, an interaction model of the crushed gangue and rock pillar in the gob bearing process is established. Regulating the density of the dry crushed filling material is crucial for ensuring the stability of the gob, providing a fundamental basis for investigating overburden and surface movement.

## Test methods and devices

### Gangue collection and preparation

**Figure 1:**
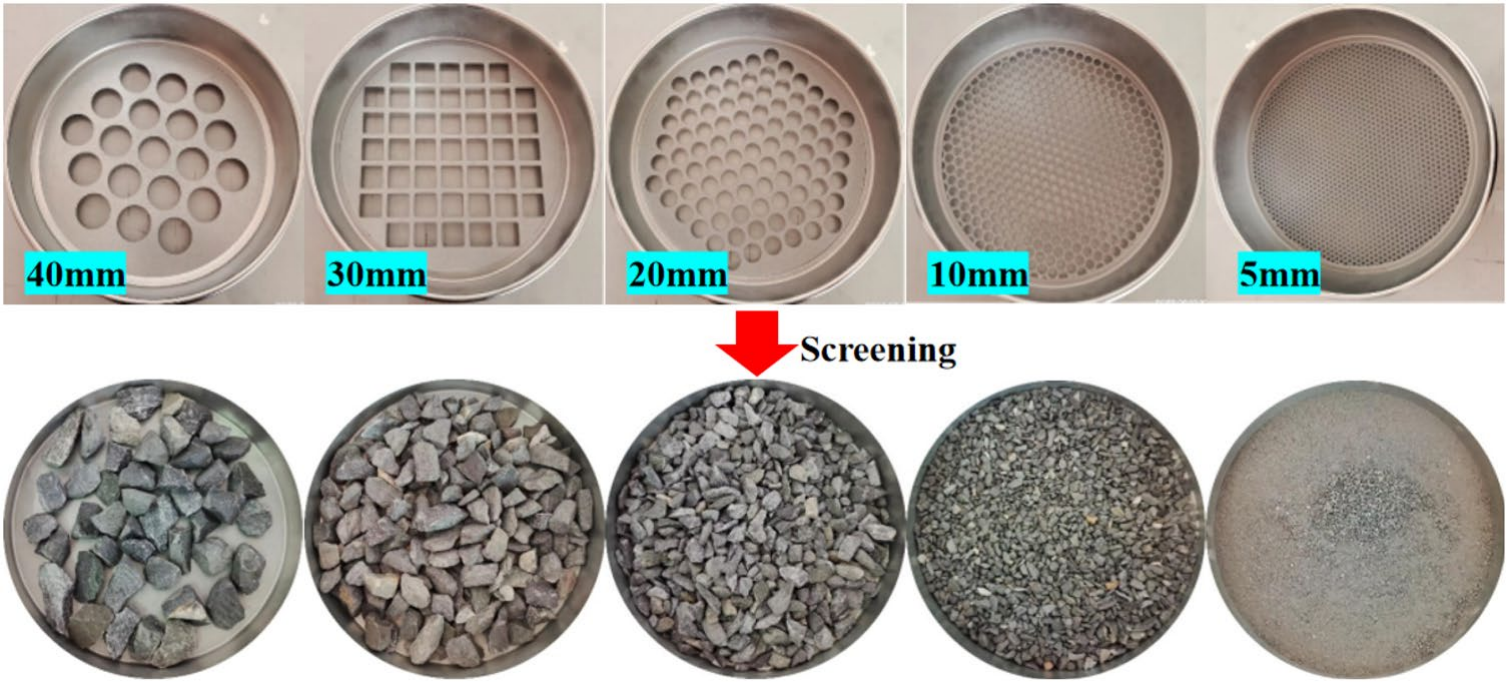
Different stone particle size screening and screening of gangue.

The material used in the test is gangue after crushing treatment, which is taken from a mine in Inner Mongolia at a depth of 400 m. Before the test, the gangue was crushed to a particle size below 40 mm by a crusher, and then closed and preserved after crushing to prevent weathering. The crushed gangue was screened by grading sieve, and the pore size was 5, 10, 20, 30, and 40 mm in turn. The used grading screen and the crushed gangue particles under different particle size ranges after screening are shown in Fig. 1.

### Experimental system and scheme

**Figure 2:**
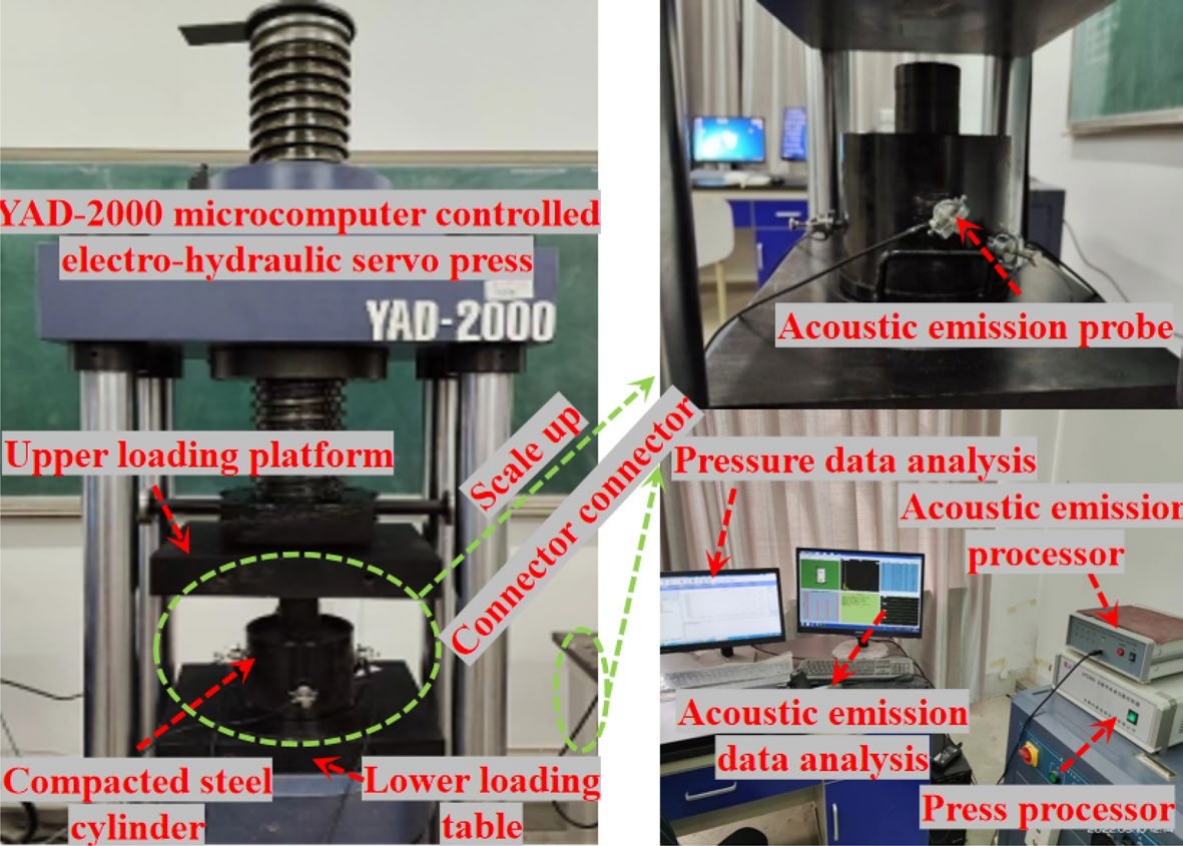
Test equipment system diagram.

YAD-2000 microcomputer-controlled electro-hydraulic servo press is the single axis press used in the waste rock particle compaction test. It is mainly composed of single-axis press, full digital electro-hydraulic servo controller and real-time data display interface. The stress and displacement of the specimen under pressure can be directly observed in real time. In order to facilitate sample loading and unloading, the size of the compacted steel cylinder is designed independently, and the horizontal displacement of the sample is controlled (the lateral constraint condition is simulated in the field filling). According to the principle that the ratio between the inner diameter of steel cylinder and the maximum particle size of dry filling material in the test is not less than 5:1^19^. The inner diameter of the steel cylinder designed for this test is 200mm and the maximum loading height is 125mm. The acoustic emission monitoring system is composed of processor, host, connector, etc. In the test preparation stage, 5 sensor probes are arranged on the outer wall of the steel cylinder at different heights to meet the spatial positioning conditions of acoustic emission. In order to avoid the collision between the waste rock and the compacted steel cylinder, an acoustic emission sensor is installed at the compacted steel cylinder. Monitor whether the sound wave signal is emitted on the surface of the compacted steel cylinder, and timely find and deal with cracks and damage on the surface of the steel cylinder. The collision between waste rock and compactioned steel cylinder is avoided and the accuracy of test data is not affected. The specific test equipment is shown in Fig. 2.

When waste rock and mixed materials fill the goaf. In general, the surrounding of the goaf is in a fixed state (this paper takes the surrounding fixation as an example), and the water in the goaf is not considered. Due to the restriction of surrounding rock and supporting structure, there is almost no displacement and deformation in the horizontal direction. In the vertical direction, the deformation is more obvious under the action of the overlying rock layer. Therefore, under the lateral nearly complete constraint and only vertical pressure, the compressive strength characteristics of gangue filling materials have been widely concerned in the practice of dry filling mining engineering. The key to improve the overall filling effect is to study the compressive strength characteristics of gangue dry filling materials and obtain reasonable particle size grading. In this experiment, Talbot theory is used to carry out the continuous particle size grading design of gangue, complete the preparation of the material for the test of gangue's compressive strength characteristics, and carry out the compaction test of gangue particles. The formula of grain size grading of gangue designed by Talbot theory is^20^ :

1 $$ P = 100 \times (\frac{d}{D})^{n} $$

where P is the passing percentage of each particle size of the bulk, %; d is the particle size in bulk, mm; D is the maximum particle size of bulk, mm; n is the gradation coefficient.

According to the Talbot theory design, the maximum particle size D set in the gangue particle compaction test is 20, 30 and 40, respectively. If the Talbot coefficient is too large or too small, it is not conducive to the actual situation of sample compression, so the Talbot coefficient is divided into 0.4, 0.6 and 0.8 levels.

## Compaction characteristics of gangue particles

### Analysis of fracture degree, stress–strain and porosity results

1. The degree of particle fragmentation
  During the preparation of the sample, waste stone particles of three colors (with a particle size of 20–40 mm) were added to analyze the crushing condition of waste stone after compaction. The stained waste rock was placed at the bottom (white), the middle (red) and the top (blue) of the sample. If the maximum particle size of the sample is D = 20 mm, the color gangue particles cannot be added and are discarded. Therefore, only six groups of samples (D = 40 mm, D = 30 mm) and a group of uniform graded samples in Talbot samples were tested for color. Before starting the loading, the body of the compacted steel cylinder is stably placed on the base, and the dispersed waste stone particles are mixed evenly. Pour into the steel cylinder in 4 times, each loading once, and smooth its surface with a piston. After the first loading and ramming, fill the bottom of the steel cylinder with white waste stone with a particle size range of 30–40 mm. After the second charge is rammed, red waste rock with a particle size range of 30–40 mm is filled in the middle of the steel cylinder. After the third charge is rammed, the top of the steel cylinder is filled with blue waste rock of the same particle size range. After loading, the piston is placed in the cylinder to flatten the specimen surface. Then the whole compacted steel cylinder containing the sample is placed on a uniaxial press for axial compression test. The loading rate is 0.5 kN/s with the test force as the test force. The initial test force assigned to the sample is 1 kN. The axial pressure is calculated according to the actual buried depth of the underground working face and the average density of the overlying strata, and the end condition of the test stress is determined to be 10 MPa. The crushing of gangue particles with three colors after the test is shown in Fig. 3.

**Figure 3:**
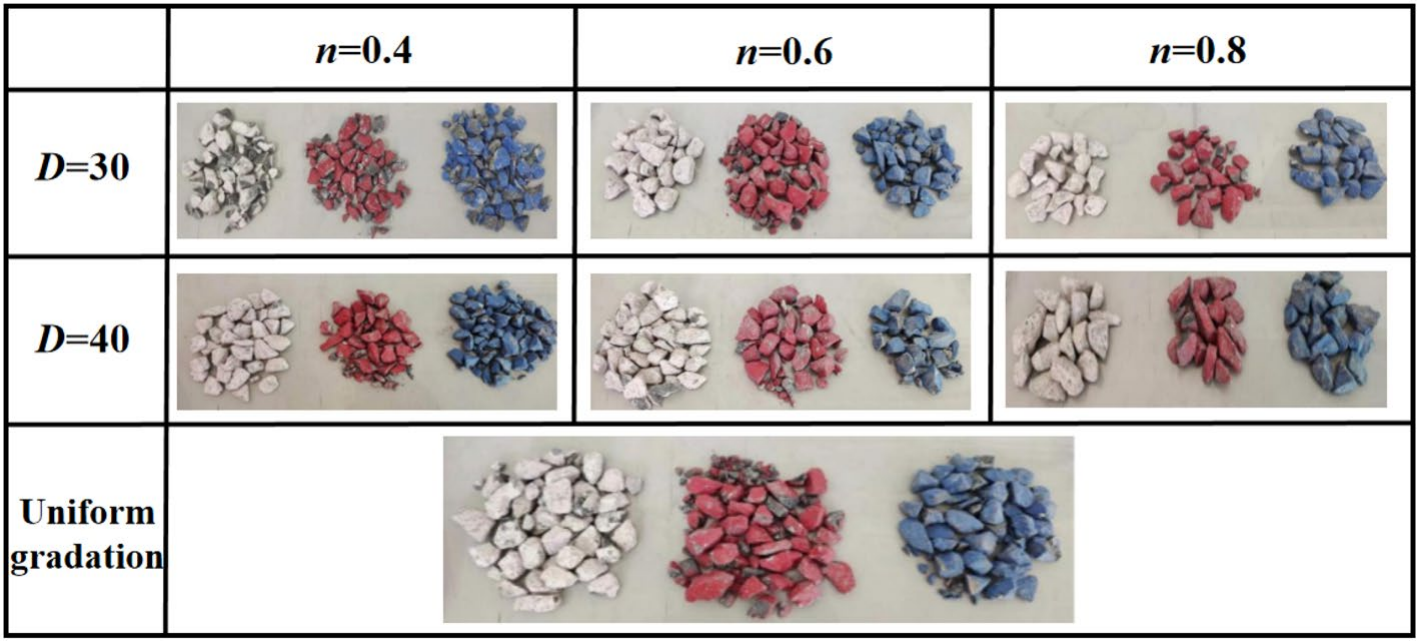
Breakage of gangue particles.
  As can be seen from Fig. 3, blue waste rock particles in each group have the highest degree of fragmentation, followed by red, and white particles are relatively intact. It can be inferred that the top waste rock particles are the first to be crushed under pressure during the test. Secondly, the axial pressure is transferred to the middle and bottom of the sample through the force chain. When the overall pressure of the sample reaches a certain degree, the inner part becomes more and more dense. When the force chain structure has a certain strength, the middle gangue particles begin to friction and roll and break, so as to improve the middle structure of the sample and enhance its stability. After the middle part was stabilized, the waste rock particles continue to roll, break and restructure until the lower part is stable.
2. stress–strain characteristics
  Based on the load and displacement data collected during the compression process of gangue particles, the corresponding stress–strain curves are obtained. The stress–strain curves of different particle sizes and Talbot coefficient are shown in Fig. 4. Figure 4 shows that:

**Figure 4:**
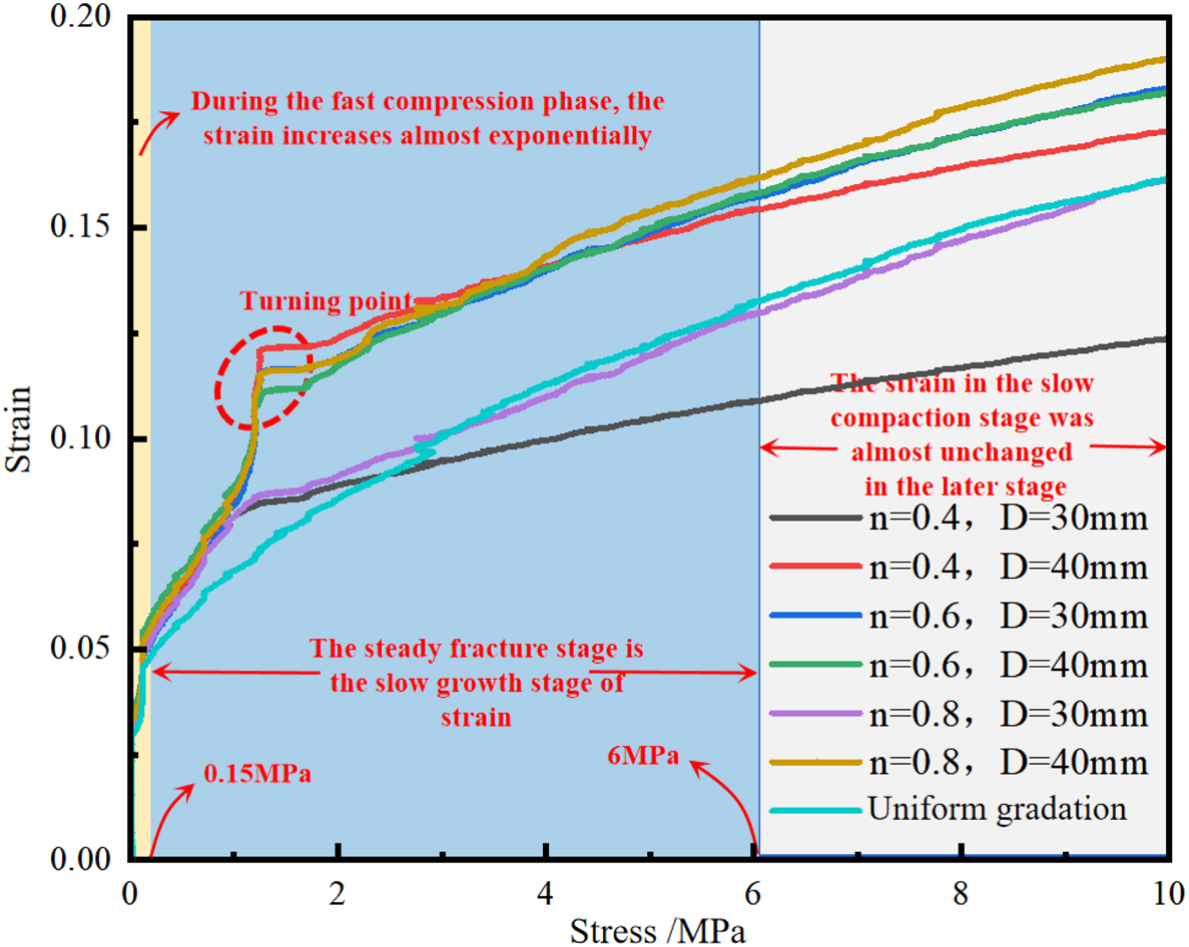
Strain–stress curves under different particle sizes and Talbot coefficients.

  - The stress–strain curves of crushed stone with different particle size and Talbot coefficient are nonlinear in the process of confined compression. The variation trend is basically similar, and the stress and strain are positively correlated. But under different stress levels, the strain change law is different. According to the evolution of the slope change rate of the stress–strain curve, the process of lateral uniaxial compression can be divided into three deformation stages^21^. The specific forms are rapid compression stage, stable crushing stage and slow compaction stage. Stage 1: rapid compression stage (0–0.15 MPa). At this stage, the axial deformation of the sample is mainly due to the direct compression of the initial pores between the particles in the sample by the axial pressure, and the large amount of friction and rolling between the particles reduces the porosity inside the sample. The axial strain accounts for more than 45% of the final axial strain. Stage 2: stable crushing stage (0.15-6mpa). The axial deformation in this stage is mainly caused by the change of gangue particles. That is, the state of direct compression changes to the state of stress concentration between particles exceeding their own strength, resulting in damage or fracture of particles. After crushing, new pores are generated, and the original pores are filled twice. In this stage, the rate of strain change was not large, accounting for about 35% of the total, and the strain growth rate gradually decreased. It is worth noting that the obvious inflection point within this stage, by analyzing the large probability is due to the fact that the particles are in an equilibrium state when they pass through a given screen (or mold) in laboratory and field stress screening tests. When a certain stress level is reached, the ability to dissipate internal energy to overcome friction and contact forces becomes increasingly difficult to maintain this steady state. Resulting in the appearance of obvious inflection points, but hardly affecting the analysis of the test data. Stage 3: slow compaction stage (6-10mpa). In this stage, the inside of the sample is gradually compacted, and the porosity is gradually reduced to a stable minimum value. The occurrence of crushing or secondary crushing of waste rock particles is gradually reduced until finally no crushing occurs. There is only a small amount of friction rolling in a small range, and its strain gradually approaches a certain value. Compared with the first two stages, it can be seen that the macroscopic deformation of the sample mainly comes from the compression and filling of the internal pores by gangue particles. In the first stage, although it is in the state of low stress, the strain produced on the sample is the most prominent.
  - There are differences in the compression deformation characteristics of gravel under different particle sizes and Talbot coefficient. Under the condition of the same maximum particle size D, the larger the Talbot coefficient of the sample, the stronger the initial compression resistance of compaction loading, and the smaller the Talbot coefficient, the stronger the deformation resistance of the sample. Mainly due to lateral limiting conditions. The internal particle structure of waste rock sample is constantly reconstituted during the process of compaction loading. With friction and fragmentation between particles, a certain amount of stored energy is released.
  - The stress–strain curve of the sample will also produce large lateral fluctuations locally due to the movement and breakage of gangue particles, and the higher Talbot coefficient is, the greater the lateral fluctuations will be. Among all the samples, the transverse fluctuations of the conventional graded samples with large particle size were the most intense during the test. It may be due to the influence of the structural frame of gangue sample, especially in the early stage of compaction loading, on the stability and support strength of the internal structure of gangue sample due to the variation of particle strength, particle size, roughness and irregularity of particle shape.

(3) strain-porosity and stress-porosity
  The porosity can directly reflect the compactness of gangue particles in the filling process. The greater the porosity, the lower the compactness of gangue particles. The calculation formula of porosity *P*_*k*_ is as follows:
  2 $$ P_{k} = \frac{{V - V_{0} }}{V} $$
  where *V* is apparent volume of gangue when loose, mL; *V*_*0*_ is a absolute volume of gangue at absolute compaction, mL.

The absolute volume of gangue cannot be measured directly (gangue is inside the steel cylinder), so the absolute density of gangue can be measured first, and then the absolute density of gangue can be obtained by calculation.

First, take the appropriate amount of water with the measuring cup, record the volume reading of water *V*_*1k*_, and then put the measuring cup containing water on the electronic scale to weigh, record the data *m*_*1k*_; at this time, add an appropriate amount of gangue, wait for it to be completely immersed and stand. When the water droplets on the edge of the cup are flowing down fully and no bubbles are attached to the surface of gangue particles, read the stable liquid level volume in the measuring cup and record it as *V*_*2k*_, and so on, record the mass *m*_*2k*_, record the measurement data, and calculate the absolute density of gangue, as shown in Eq. (3) :

3 $$ \rho_{0} = \sum\limits_{k = 1}^{{}} {\frac{{m_{2k} - m_{1k} }}{{V_{2k} - V_{1K} }}} $$

According to the measured data, Table 1 is drawn:

**Table1:** Waste rock absolute density calculation test data.

*K*	1	2	3	4	5	6	7	8	9	10
*V*_*1k*_ (ml)	500	500	600	600	700	700	800	800	900	900
*V*_*2k*_ (ml)	540	530	640	628	730	746	815	832	915	938
*m*_*1k*_ (g)	1125	1125	1225	1225	1325	1325	1425	1425	1525	1525
*Ρ*_0_	2.850	3.267	3.150	2.714	3.100	3.217	2.867	4.031	3.133	2.868

In order to reduce measurement errors, the above weighing process was repeated ten times, and the absolute density of gangue was calculated according to Table 1 as follows:

4 $$ \rho_{0} = \frac{1}{10}\sum\limits_{k = 1}^{10} {\frac{{m_{2k} - m_{1k} }}{{V_{2k} - V_{1K} }}} = 3.120g/cm^{3} $$

The inner radius of the steel cylinder is set as *r*, the residual height of gangue after the test is *h*, and the mass m of gangue does not change before and after the test.

5 $$ V = \pi r^{2} \cdot h $$

6 $$ V_{0} = \frac{m}{{\rho_{0} }} $$

Simultaneously Eqs. (3) to (6) can be obtained:

7 $$ P_{{\text{k}}} = 1 - \frac{m}{{\rho_{0} \pi r^{2} h}} $$

Assuming that the initial loading height of gangue is *h*_*0*_, the axial strain of gangue sample in the process of lateral limited uniaxial compression can be calculated as follows.

8 $$ \varepsilon = \frac{{h_{0} - h}}{{h_{0} }} = 1 - \frac{h}{{h_{0} }} $$

Simultaneously Eq. (7) to Eq. (8) can be obtained:

9 $$  P_{k} = 1 + \frac{m}{{\rho_{0} \pi r^{2} (\varepsilon - 1)h_{0} }} $$

It follows that when *h*_*0*_ is a constant value, the porosity *P*_*k*_ decreases with the increase of axial strain. When the strain is 0, the initial porosity increases with the increase of the initial charge height *h*_*0*_, which is positively correlated.

The weight of seven groups of gangue samples used in the test is all 5 kg, and the initial charging height is all 12 cm. Given the inner diameter and height of the steel cylinder, the porosity corresponding to different axial stresses can be calculated. Since the axial strain is a proportional function of the axial stress during lateral confined axial compression, the porosity is expected to decrease as the axial stress increases. The stress–strain curve of gangue is fitted, and the fitting relationship is obtained as follows.

10 $$  \varepsilon = a + b \times ln (\delta + c) $$

According to Eqs. (7) and (10), the relationship between porosity and axial stress can be obtained as follows:

11 $$  P_{k} = 1 + \frac{m}{{\rho_{0} \pi r^{2} h_{0} \left[ {a - 1 + b \times ln (\delta + c)} \right]}} $$

**Figure 5:**
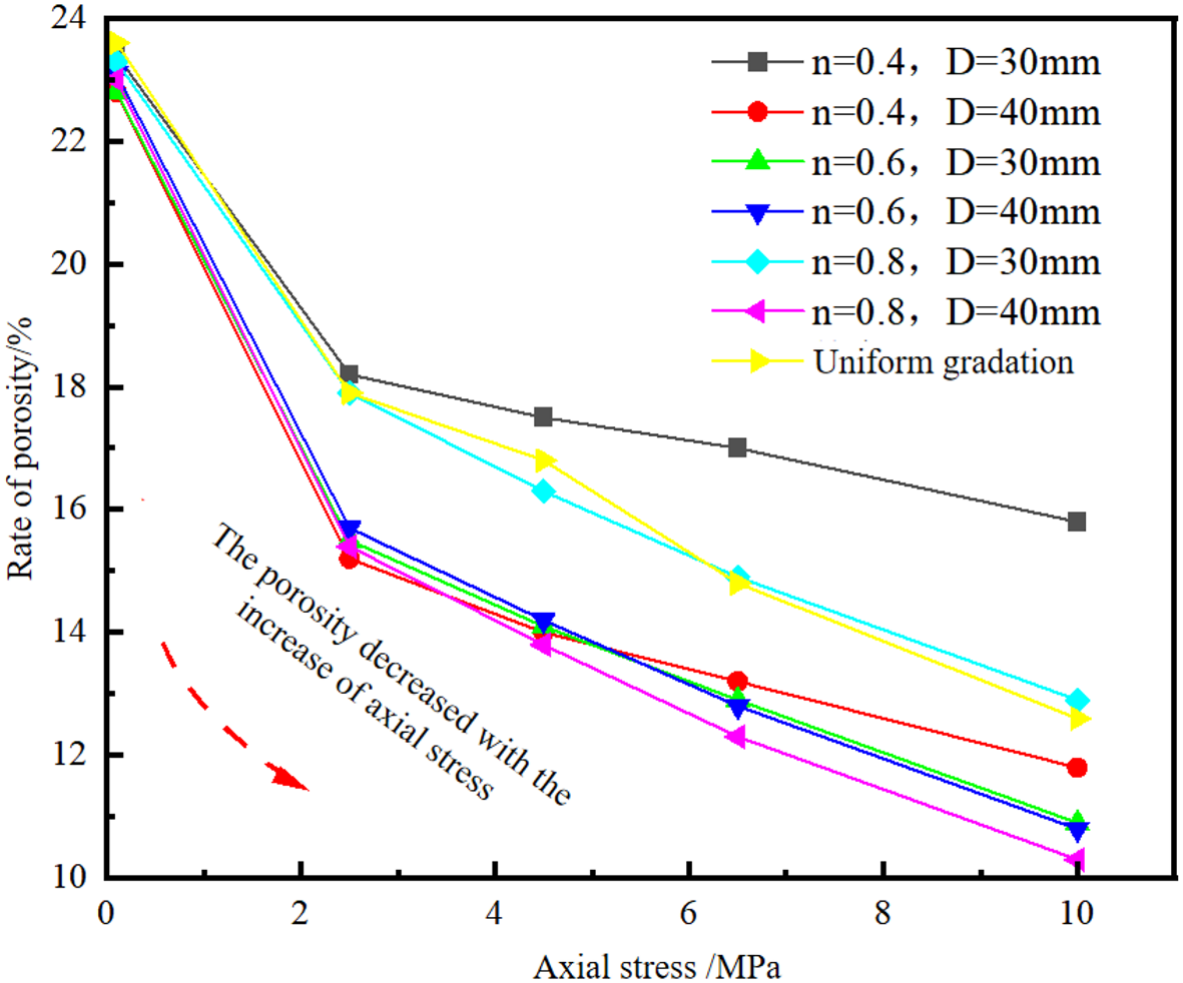
Axial stress-porosity curve.

The axial stress-porosity curve drawn according to the axial displacement data obtained in the test is shown in Fig. 5.

Figure 5 shows that the relationship between porosity and axial stress is generally monotonically decreasing, and the decreasing rate is gradually decreasing. The test results are consistent with those derived from Eq. (11). The samples with uniform gradation had higher porosity than those with other gradations, and the final porosity of the large particle size gradation was the lowest. Therefore, the strength of the pressure bearing structure inside the sample is not determined by the proportion of large particle size particles in the sample, but is affected by the maximum particle size D, the grading coefficient n, and the continuity of particle size distribution. The higher the Talbot coefficient of the sample, the lower the final porosity. At the same time, the pressure bearing structure composed of large and small particles inside the sample supports the external axial pressure and the sample's own gravity together. The lower the final porosity, the higher the bearing strength and stability of the structure inside the sample, and the better the filling effect.

### AE event spatial anchor point for axial compression of gangue particles

**Figure 6:**
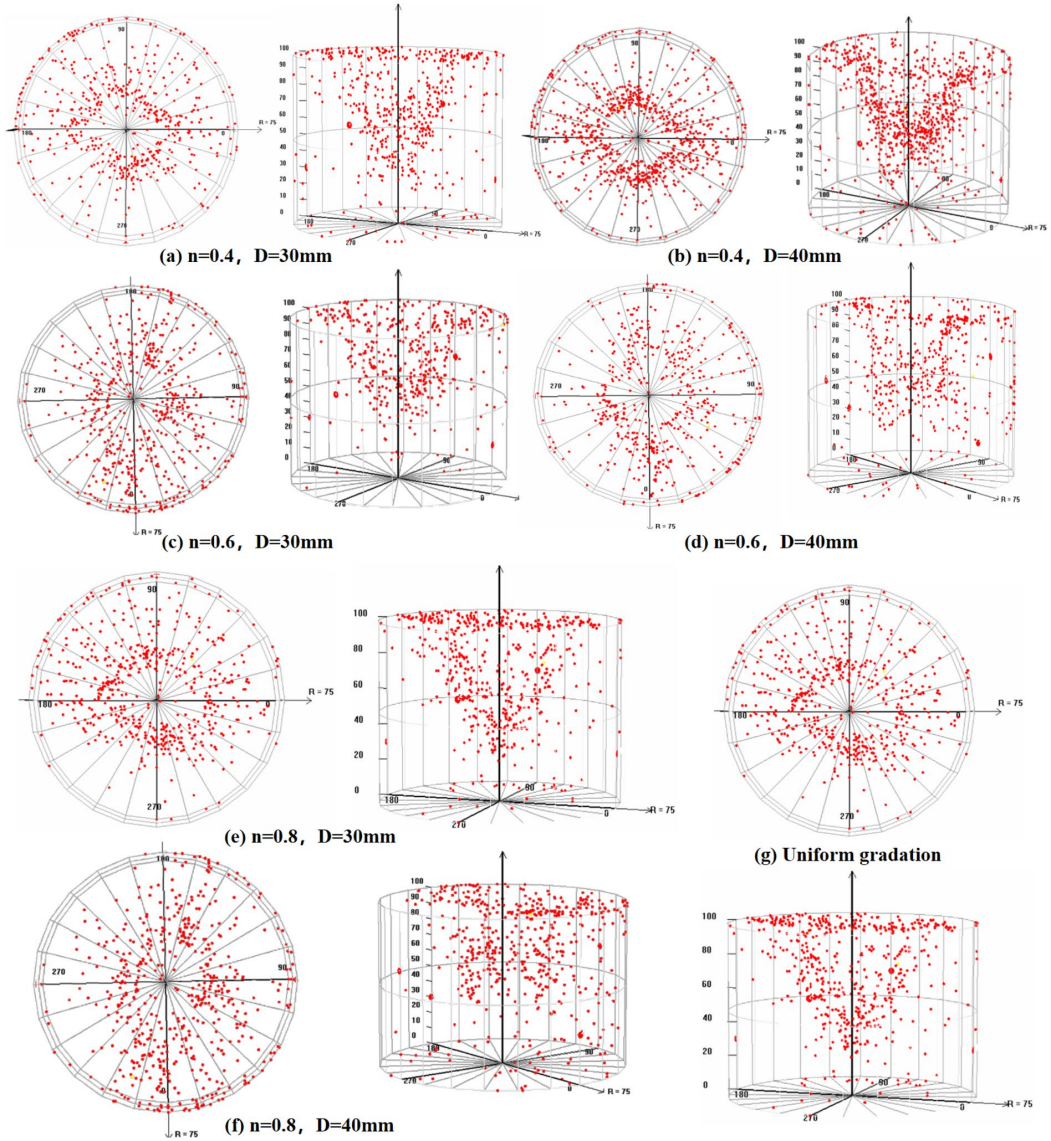
AE event spatial anchor point map of gangue with different particle size grades.

The AE system can determine the specific location of internal damage and breakage of the sample by monitoring AE events in the sample. During AE event monitoring, it can synchronize with the above sensors to observe whether there are abnormal phenomena. In addition, a series of benchmark tests are conducted on gangue particles to record AE event characteristics under normal conditions to ensure the accuracy of AE events. Figure 6 shows the AE events inside the sample after compaction deformation and the spatial location map in the 3D diagram,

Figure 6 shows that the AE events of Talbot sample show a conical shape on the spatial anchor point map, and the more obvious AE events are mainly concentrated in the middle and upper parts of the sample, with the feature of stepwise concentration. Therefore, in the process of lateral limiting axial compression of the sample, the gangue particles with a large influence range are mostly broken in the middle and upper parts of the sample, which is consistent with the colored gangue particles broken observed in 1.3 above. Therefore, it can be concluded that there is a stepwise centralized transmission of axial pressure in the sample, and the influence range of breakage gradually decreases from top to bottom. In the conventional graded samples, the AE events of large and small particle sizes did not appear obvious concentration phenomenon, and were relatively dispersed, but it can be seen that there are still more AE events in the middle and upper parts of the sample, and there is a hierarchical phenomenon. It may be due to the defects in particle size grading of large and small particle sizes of the sample, there is no continuity, and the force chain structure inside the sample is stable. The axial pressure during the test was not transmitted downward in a centralized manner, but diffusely and outwards. Therefore, the spatial distribution of AE anchor points detected by AE was relatively scattered. The top view of the AE event localization shows that the gangue particles have a tendency to move laterally and outwards during axial compression of the sample. At the same time, the gangue near the cylinder wall also has a lateral inward movement trend due to the side limiting effect of the steel cylinder, resulting in a relative tangential extrusion, especially at the middle position from the cylinder wall to the center of the circle, the crushing situation is the most obvious, which is consistent with the research results of Xiao Bo et al^21^ on the internal damage and the specific location of the breakage of the sample. It should be noted that the shape formed by the top view of the AE event space positioning point map obviously has a "hollow", while there is a "conical" cusp in the front view map. The possible reason is that AE events occur in some openings, holes or areas with wide cracks inside the material, which may present a circular hollow state when viewed from above. However, when viewed from the side, the hollow shape may take on a conical shape due to space limitations.

### Acoustic emission signal ringing counting-axial stress–strain–time relationship

**Figure 7:**
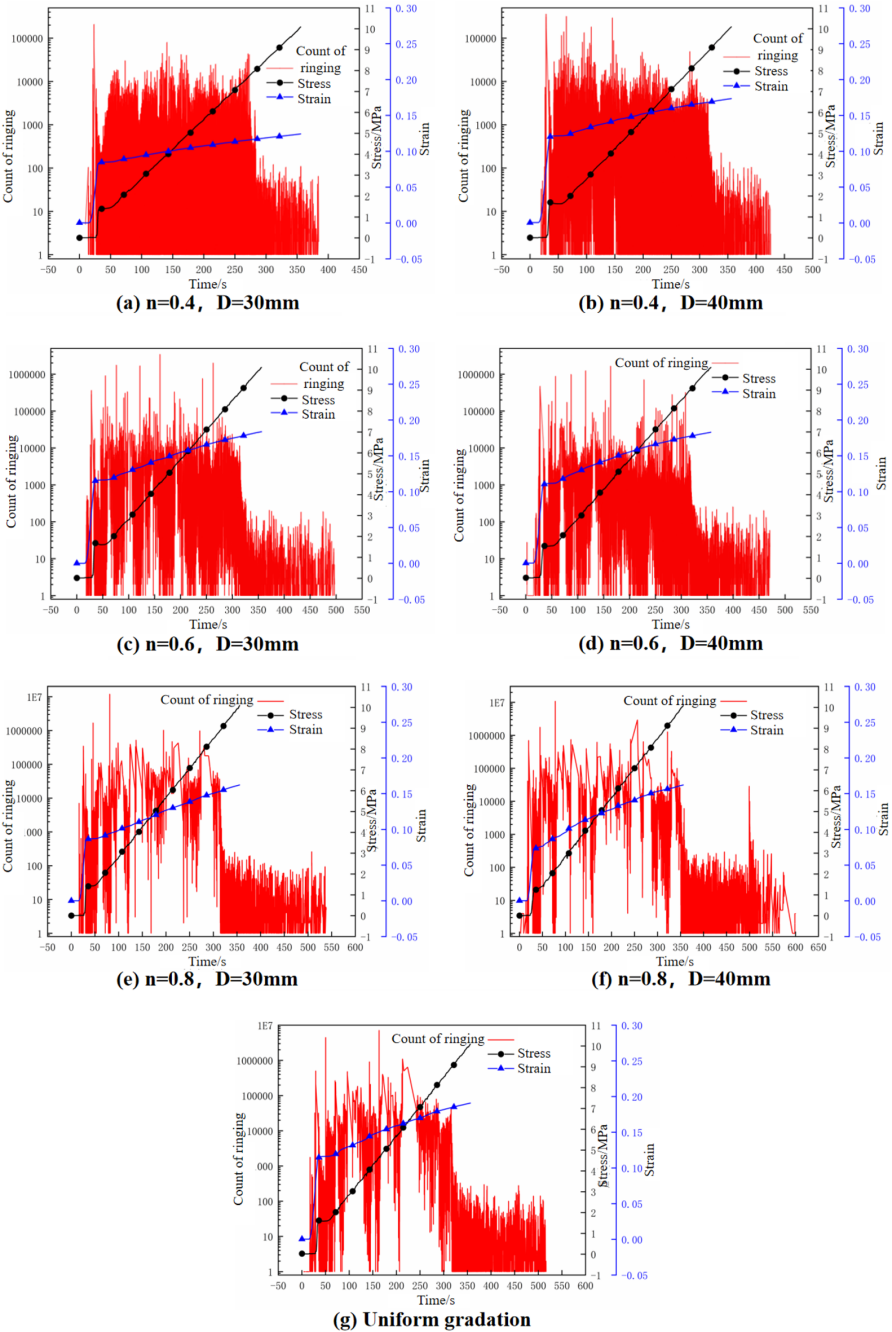
Acoustic emission ring counts of specimens during confined axial compression.

According to the test data generated by AE equipment, the relation between AE signal ringing count, axial stress–strain–time in the lateral confined axial compression process of gangue samples is obtained as shown in Fig. 7.

The ringing count of AE signal can characterize the occurrence frequency of breakage phenomenon inside the sample to a certain extent. Figure 7 shows that the acoustic emission ringing count of gangue in the process of lateral limiting axial compression is roughly divided into three stages:

- In the pore compaction stage (corresponding to the rapid compression stage): In the early stage of loading, the pores among gangue particles are large, and the connection between gangue particles with different particle sizes in a single sample is not close. When the axial load is small, a majority of particles in the sample will roll, slide, and shift under the influence of the Z-axis load, and even a small number of low-strength particles may rupture and undergo structural reorganization. During this stage, the axial strain increases sharply, with the highest growth rate observed throughout the entire loading process. At this point, there are fewer occurrences of AE ringing counts, and their values are low, indicating a low degree of gangue particle breakage during this stage. The AE ringing count value is influenced by the rapid rise in axial strain at this stage.
- In the crushing compaction stage (corresponding to the stationary crushing stage): With the continuous confined compression, the axial stress and strain increase continuously, and the stress concentration is significant at point-point or point-surface contact between waste rock particles. A large number of waste rock particles are gradually broken. In this stage, the acoustic emission ringing count shows a jump change around a high level value. The reason is that a large number of gangue particles break in the sample at this stage, and the overall breakage degree of the sample increases. After the initial loading, the axial stress increases at a constant change rate due to the loading rate set by the test, while the axial strain decreases continuously in the process of continuous loading and finally approaches a certain value, which is consistent with the results of the crushing mechanism of crushing gangue in the compression process studied by scholars like Le Zhihua et al.^22^ through acoustic emission.
- In the compaction and consolidation stage (corresponding to the slow compaction stage): since the crushed gangue particles fill the remaining void, the stress concentration between gangue particles is relieved to a certain extent. But the sample is still in the axial compression state of the lateral limit. In the second stage, the gangue particles that have not broken will rupture under the increasing axial stress. Even the broken gangue particles continue to break twice or more, so the AE ringing count remains at a high level in the early stage of the stage. At this stage, the AE ringing count began to decrease and eventually stabilized at a low level, indicating that the internal sample had entered a stable compaction stage. However, for the large and small particle size samples with conventional gradation, the AE ringing count started to decrease to a low level when the axial stress curve ceased to grow (i.e., when the test stopped loading). This observation suggests that the discontinuously graded sample continued to experience crushing and compaction during the later stages of axial loading, leading to a significant breakage of gangue particles.

According to the above analysis, with the increase of stress, the evolution characteristics of acoustic emission parameters of broken rock show a phased change, and show a similar evolution law. The evolution process of acoustic emission corresponds to the compaction process of broken specimens.

### Acoustic emission energy characteristics of dry filling materials under axial compression

**Figure 8:**
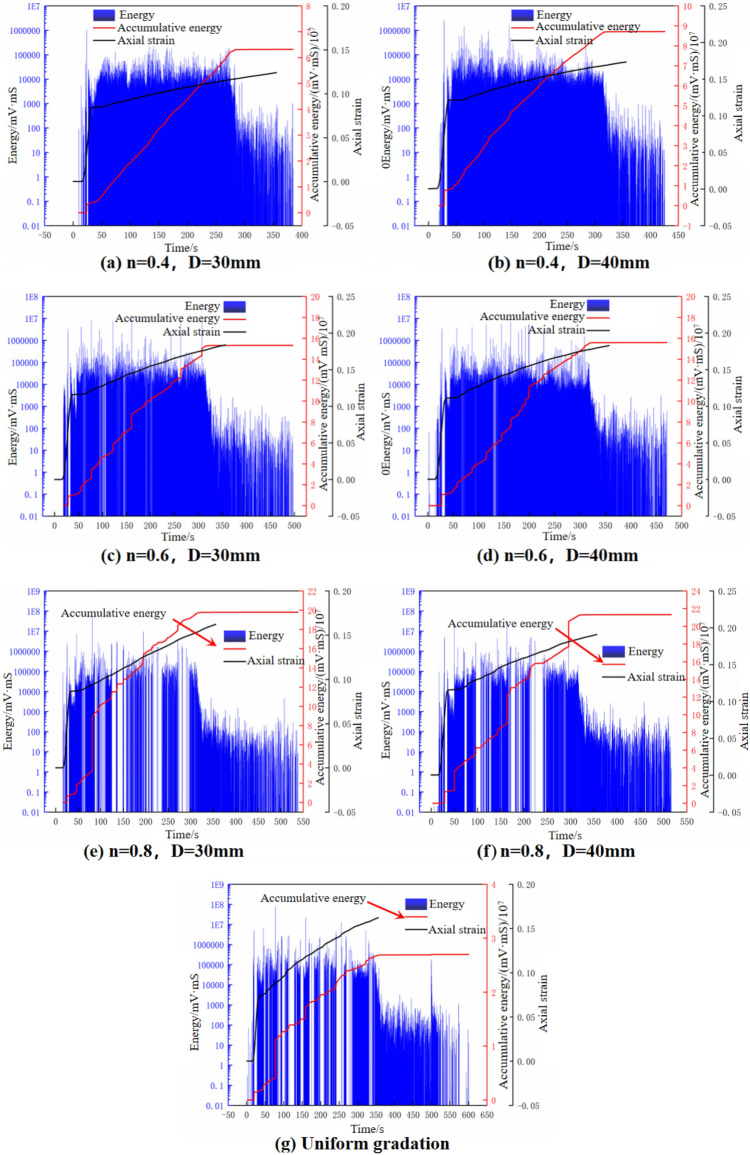
AE energy-cumulated energy-axial strain–time curve of gangue samples.

The data obtained from the AE monitoring system was used to draw the characteristic curve of the relationship between axial strain-AE energy-cumulative energy-time during the axial compression process of gangue samples with different particle size grades, as shown in Fig. 8.

Figure 8 shows that the cumulative energy-time curve of gangue sample can be divided into three stages in the overall change trend in the process of lateral confined axial compression:

- In the early stage of loading (corresponding to the rapid compression stage): There are few AE signals in this stage, and the energy intensity is not high, and the growth rate is small. This may be due to the fact that the gangue particles in the sample at this stage are mostly frictional rolling, misalignment and pore filling, and the axial pressure is small, which is difficult to cause the rupture between the particles. Therefore, the AE signals were mainly derived from rolling friction AE events. The energy intensity generated by the AE signals was not high, and the cumulative energy curve grew slowly.
- In the middle loading stage (corresponding to the stable crushing stage): During this stage, the accumulated energy signal exhibits a cliff-like rise and continues to evolve at a relatively moderate growth rate. This could be attributed to the gradual compaction of the sample's interior as the axial stress steadily increases. Initially, in the early stages, the rolling friction among particles and the filling of pores establish a relatively stable force chain structure, which initially provides a certain level of bearing strength. At the middle stage, due to the large number of pores in the sample, the axial pressure can not be well transmitted, and the stress between the particles is concentrated. The particles release the concentrated energy through the form of rupture, and the influence range is wide, until the rolling friction continues to occur around. Therefore, there are a lot of particle crushing and friction rolling in the middle stage. In comparison to the energy produced by frictional rolling, the energy generated by gangue particle crushing experiences a significant geometric increase, leading to a steep rise in the cumulative energy curve. Once it reaches a certain threshold, the energy curve begins to gradually elevate at a higher level. During the intermediate stage, the AE signals comprised of events related to rolling friction and broken recombination, providing additional insights into the observation of a sudden development in energy intensity around higher levels.
- In the late loading stage (corresponding to the slow compaction stage): Under the influence of continuous high-strength axial pressure, the sample undergoes cyclic structural reorganization and tends to stabilize, while reaching a peak in terms of density. Although the stress concentration between particles is higher compared to the intermediate stage, the presence of fewer pores in the sample allows for predominantly axial transmission of pressure. As a result, friction and rolling between particles continue to occur during the later stage. At this stage, the AE signal is mostly rolling friction AE events, and the energy intensity gradually decreases to a stable low level. The growth rate of cumulative energy intensity is also close to zero due to the small growth range, making the curve approximately parallel development.

## Compressive mechanical response mechanism of crushed gangue bearing in gob

**Figure 9:**
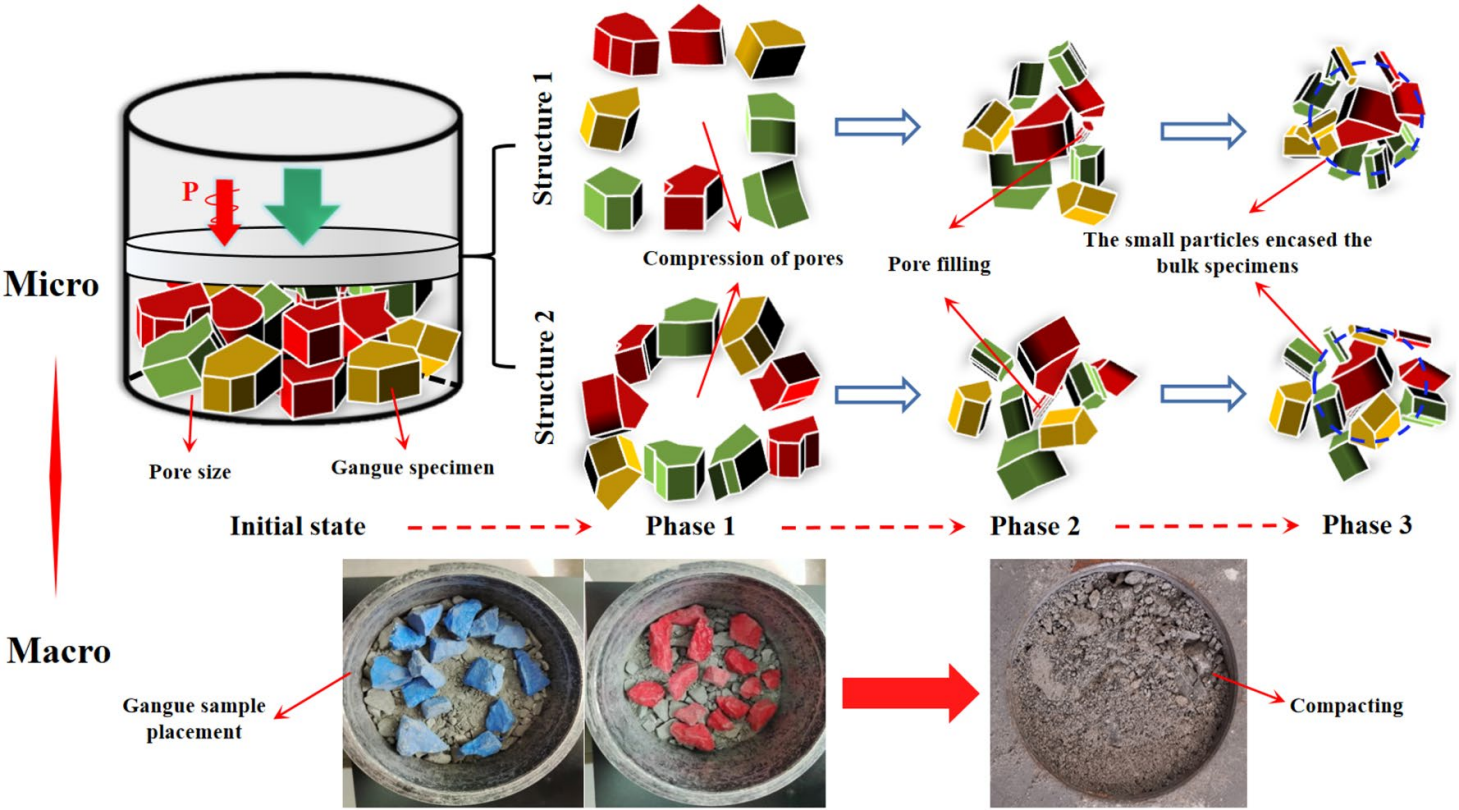
Deformation mechanism of broken gangue based on mesoscopic fabric.

Combined with the previous research results of stress–strain, porosity, breakage degree and acoustic emission parameters of broken samples, the deformation mechanism of crushed gangue as filling material in the process of lateral confined compression load is revealed from a mesoscopic perspective. According to the above analysis, the deformation process of crushing gangue in the compression process is divided into three stages, as shown in Fig. 9.

**Figure 10:**
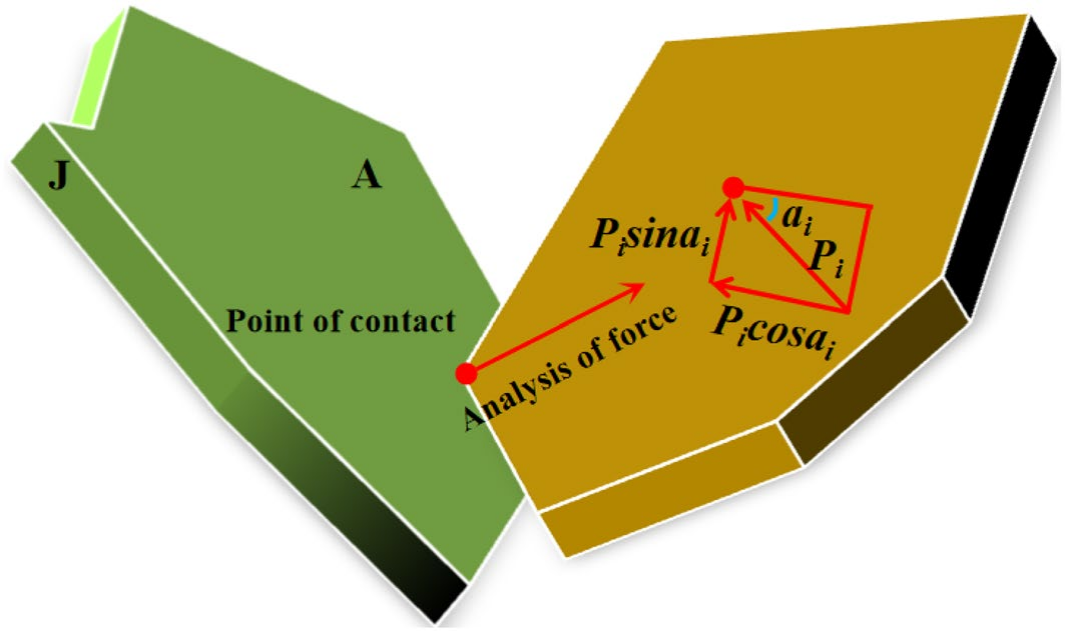
Force analysis diagram of crushed waste rock.
  In the axial stress loading process, if the *K* loading stage is applied to the crushing gangue, the corresponding axial load is *F*_*K*_ (K = 0,1,2,… i, j, j + k,…) When the force *F* ≤ *F*_*i*_, *δ*_*A*_ is small, which is far less than the medium strength of crushed gangue, and the gangue particles almost do not crush and break. At this point, the particle state is predominantly influenced by shear force, leading to the overcoming of friction resistance between particles. This results in a significant amount of rotation, sliding, and dislocation, which greatly reduces the pores between particles, causing substantial axial deformation of the sample. The contact between particles becomes denser, and through continuous reorganization, the internal structure of the sample attains a degree of stability and compressive capacity. Consequently, the rate of axial strain growth begins to decline.
- Second phase: during the stable crushing stage, the deformation of the gravel gradually slows down. This slowdown occurs primarily because the axial deformation within the sample is directly compressed by the state of the gangue particles, leading to stress concentrations between the particles that exceed their own strength states (*F*_*i*_ < *F* ≤ *F*_j_), which leads to the breakage or fracture of the particles. However, when the axial load exceeds *δ*_*cr*_ (the compressive strength of crushed gangue), δsp (the strength of angular and edge local damage), or *δ*_*i*_ (the contact extrusion pressure) exceeds the bearing force of most gangue, the local breakage of angular and edge will occur at *K*_*1*_ positions, and the overall rupture of *K*_*2*_ particles will occur. The expression is as follows:
  13 $$  \delta_{i}^{{k_{1} }} \ge \delta_{cr}^{{k_{1} }} \,\, (k_{1} = 1,2, \ldots ,K_{1}) $$
  14 $$  \delta_{i}^{{k_{2} }} \ge \delta_{sp}^{{k_{2} }}\,\, (k_{2} = 1,2, \ldots ,K_{2}) $$
  At this time, the gangue skeleton structure will be destroyed, resulting in a large number of particles in the gangue secondary fragmentation, such as fragmentation and angular fragmentation. With the increase of axial load, the compression deformation of the broken rock increases sharply until it is broken, after which the particles will produce new pores, thus filling the original pores twice and enhancing the stability of the bearing skeleton.
- The third stage is the slow compaction stage. During this phase, the specimens essentially develop a stable load-bearing skeleton structure, with the smaller broken particles encapsulating the larger sample. This enhances the stability of the load-bearing skeleton and the sample's resistance to deformation. Simultaneously, due to this phenomenon, internal stress concentration is reduced, making it difficult for the larger sample enveloped by smaller particles to fracture again.

- The first stage: the rapid compression stage, in which the rate of strain change with stress is the fastest. Suppose that the Nth particle of crushing gangue has N contact points with other particles than itself. If the crushing gangue is loaded with axial stress, the force acting on the ith contact point is *P*_*i*_ (i = 1, 2, 3 … n), the contact area between particle *N* and contact point *i* is *S*_*i*_, and the stress analysis of crushed gangue is shown in Fig. 10. Taking the stress analysis particles as the research object, suppose that there are m contact points on the contact surface A that can contact with other contact points (m ≤ n), then the normal stress and shear stress on the contact surface A are:
  12 $$  \delta_{A} = \sum\limits_{i = 1}^{m} {\frac{{P_{i} }}{{\delta_{i} }}} cos\alpha_{{\text{i}}}, \quad  \tau_{A} = \sum\limits_{i = 1}^{m} {\frac{{P_{i} }}{{\delta_{i} }}} sin\alpha_{i} $$
  where *α*_*i*_ is the angle between the parallel direction of the contact surface and the acting force;

## Mechanical model of interaction between crushed gangue and surrounding rock pillars in gob

From the above research, it can be understood that the deformation mechanism of crushed waste rock as filling material in the process of confined compression load, and the force acting on the rock column has a certain relationship with the particle size of the filling material and the Talbot coefficient. The particle size of the filling material will affect the internal pore structure and density, and thus affect the supporting pressure exerted by the filling material on the rock column. The Talbot coefficient reflects the structural characteristics of the particle arrangement inside the filling material. Filling materials with different Talbot coefficients also have different supporting effects on rock columns. In general, filling materials with a higher Talbot coefficient may form a tighter structure during the filling process, provide more uniform and stable support, and have a more significant protective effect on rock pillars. The next step is to further understand how filling materials interact with the surrounding rock pillars and enhance the overall compressive strength to make the gob stable^23^. Based on the experimental results, a mechanical model is established to describe the relationship between the particle size of the filling material, Talbot coefficient and the force acting on the rock column, so as to provide guidance for engineering practice.

**Figure 11:**
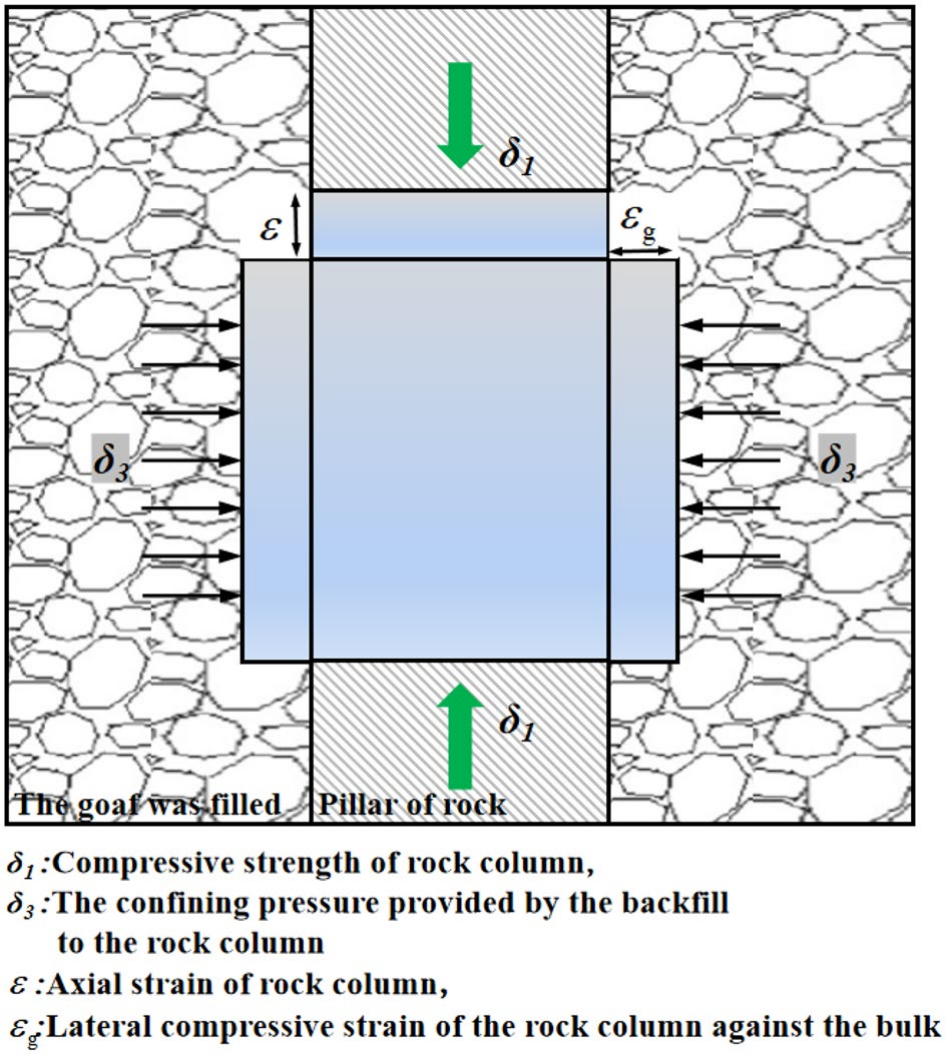
Load bearing mechanical model of dry crushed gangue filled with rock pillar.

In the process of compaction of crushed waste rock, the whole test process can be regarded as the bearing system of rock column under the condition of lateral limit when the waste rock is filled. Figure 11 shows the load-bearing mechanical model of dry crushing gangue filled with rock pillar. The assumption conditions of the model are as follows: ① the gob is completely topped when gangue is filled, and the forces of the overlying rock layer are all concentrated in the rock pillar; ②The elastic deformation of the rock column after filling with crushed gangue is only subjected to lateral compression; ③The rock column is in ultimate stress equilibrium. At this time, the expression of rock pillar stress and expansion lateral strain is ^24^ :

15 $$  \delta_{1} = \frac{2ccos\phi }{{1 - sin\phi }} + \frac{1 + sin\phi }{{1 - sin\phi }}\delta_{3}, \quad  \varepsilon_{g} = \frac{1}{E}\left[ {\delta_{3} - \nu (\delta_{3} + \delta_{1} )} \right] $$

where $\phi$ is the internal friction angle, *c* is the rock cohesion, *E* is the rock elastic modulus, and $\nu$ is the rock Poisson ratio.

Considering the active and passive confining pressure of the crushed gangue filled with its own gravity on the rock column, the confining pressure provided by the filling can be expressed as^25^ :

16 $$ \delta_{3} = \delta_{3a} + \delta_{3p} = \frac{b\gamma }{{2tan\phi }}\left[ {1 - exp(\frac{ - 2KHtan\phi }{b})} \right] + E_{g} \varepsilon_{g} $$

where $\delta_{{3{\text{a}}}}$ and $\delta_{{3{\text{p}}}}$ are the active confining pressure and passive confining pressure of rock pillar after crushing gangue filling; $\gamma$ is the bulk density of crushed gangue after filling; *b* is the filling width of crushing gangue;* H* is the filling height of crushing gangue; $\phi$ is the surface friction angle of rock pillar and crushed gangue after filling; *K* is the coefficient of lateral pressure; *E*_*g*_ is the deformation modulus of the backfill.

In the actual production process, the active confining pressure of the filling body to the rock pillar in the gob is constant, so that $\delta_{{3{\text{a}}}} = C$; And the c and $\phi$ of rock are also constants, let $A = \frac{2ccos\phi }{{1 - sin\phi }}$, $B = \frac{1 + sin\phi }{{1 - sin\phi }}$, and substitute them into Eq. (16) to obtain:

17 $$ \delta_{1} = A + B(c + E_{g} \varepsilon_{g} ) $$

Substituting Eq. (15) into Eq. (17) yields:

18 $$ \delta_{1} = \frac{{E(A + BC) + BE_{g} (1 - \nu )\delta_{3} }}{{E + BE_{g} \nu }} $$

**Figure 12:**
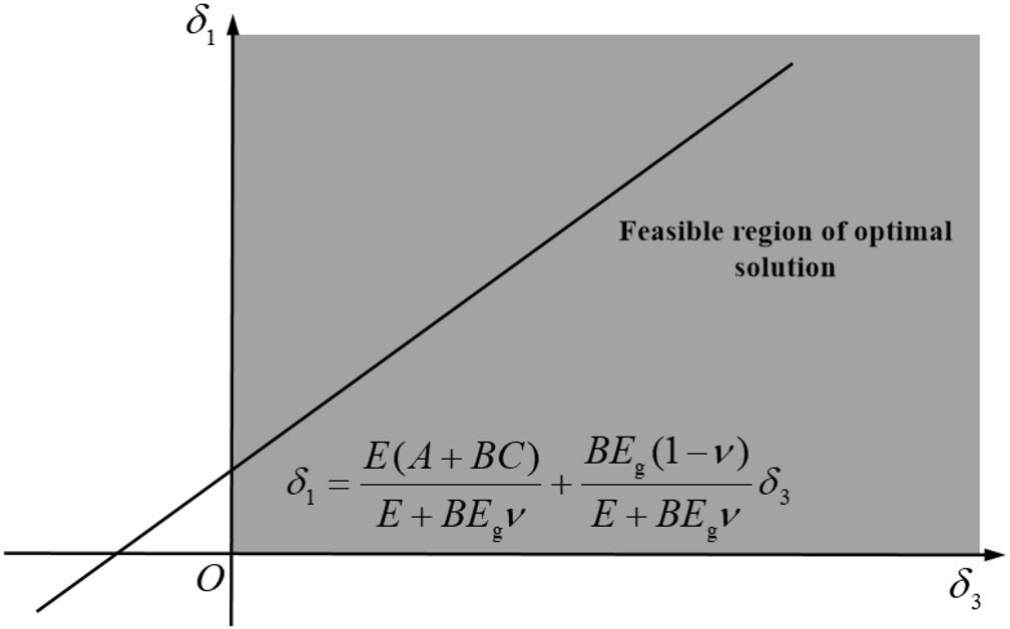
The feasible region of $\delta_{1}$ and $\delta_{3}$.

According to Eq. (18), the relationship between $\delta_{1}$ and $\delta_{3}$ is linear, and both are greater than 0, and the constants $A,B,C,E,\nu$ are also greater than 0, $E_{{\text{g}}}> 0$. The optimal solution of Eq. (18) can be obtained by mathematical calculation of variables, as shown in Fig. 12. Figure 12 shows that $\delta_{1}$ and $\delta_{3}$ is proportional, and the magnitude depends on the slope k. If the $k \ge n(n>0)$ growth rate boundary condition is assumed, Eq. (19) is satisfied with $E_{{\text{g}}}$ and $E$.

19 $$ k = \frac{{BE_{g} (1 - \nu )\delta_{3} }}{{E + BE_{g} \nu }} \ge n $$

20 $$ E_{g} \ge \frac{n(1 - sin\phi )}{{(1 - \nu - n\nu )(1 + sin\phi )}}E = \frac{n(1 - sin\phi )}{{(1 - \nu - n\nu )(1 + sin\phi )}}E $$

For Eq. (20) to hold, then $1 - \nu - n\nu > 0$, therefore, Eq. (19) needs to be satisfied $0<n<1 - \nu /\nu$_._

The stiffness of crushed gangue after filling compaction is the key to maintain the stability of gob. The essence of backfilling mining is to replace the original ore body with different types of filling materials. If $E_{g} \ge E$, there will be no instability problem in the gob, but considering the actual production process, the stiffness of crushed gangue after filling cannot be greater than the ore body (infinite approach), so, $E_{{\text{g}}}$ and $E$ meet

21 $$ \frac{(1 - sin\phi )E}{{(1 - 2\nu )(1 + sin\phi )}} \le E_{g} \le E $$

When the dry filling material is used to fill the gob, the better the bearing characteristics of rock column are $E_{{\text{g}}}$ and $E$ . According to Eq. (21), the $E_{g}$ is related to the compaction degree. When the dry filling material is used in the field, the density of the dry filling material can be controlled according to the relationship between the compaction characteristics of the material and the bearing characteristics of the material analyzed above, so that the gob tends to be stable.

## Conclusions

1. The lateral confined compression characteristics of dry filling materials with different particle sizes and Talbot coefficient are studied, and the compression deformation characteristics of broken waste rock in gob and the evolution law of slip and rupture in the compaction process of broken waste rock are obtained.
2. By integrating the acoustic emission signal ring count and the cumulative energy-time curve, the microscopic perspective revealed the deformation mechanism of crushed gangue as a filling material during lateral limited compression loading. The compression and deformation of each stage occurred synchronously, with a one-to-one correspondence in time.
3. Through acoustic emission monitoring and mechanical model establishment, the lateral confined compression characteristics of dry filling materials with varying particle sizes and Talbot coefficients were examined. This led to the determination of the compression deformation characteristics of crushed gangue in the gob and the evolution law of slip and rupture in the compaction process of crushed gangue. It was confirmed that dry crushing filling materials are crucial in gob filling for coal mines.
4. An interaction model of the crushed gangue and the rock pillar in the gob during the bearing process was established, yielding the relationship between the deformation modulus of the two. This has significant implications for guiding the enhancement of overall compressive strength and promoting gob stability through the use of dry crushing and filling materials.

## Data Availability

The datasets used and/or analysed during the current study available from the corresponding author on reason able request.
